# Association between Aspartate Aminotransferase-to-Platelet Ratio Index and Hepatocellular Carcinoma Risk in Patients with Chronic Hepatitis: A Meta-Analysis of Cohort Study

**DOI:** 10.1155/2019/2046825

**Published:** 2019-11-13

**Authors:** Chuanmeng Zhang, Jiayuan Wu, Juan Xu, Jie Xu, Jianchun Xian, Shanshan Xue, Jun Ye

**Affiliations:** ^1^Department of Central Laboratory, Taizhou People's Hospital, Taizhou, 225300 Jiangsu Province, China; ^2^Clinical Research Center, The Affiliated Hospital of Guangdong Medical University, Zhanjiang, 524001 Guangdong Province, China; ^3^Clinical Laboratory, Taizhou People's Hospital, Taizhou, 225300 Jiangsu Province, China; ^4^Department of Infectious Disease, Taizhou People's Hospital, Taizhou, 225300 Jiangsu Province, China

## Abstract

**Background and Aim:**

Aspartate aminotransferase-to-platelet ratio index (APRI) is widely used in the assessment of fibrosis and cirrhosis, especially in patients with chronic hepatitis. However, the prognostic value of APRI in patients with chronic hepatitis with regard to the prediction of hepatocellular carcinoma (HCC) occurrence remains controversial. The objective of this meta-analysis is to investigate the association between APRI and HCC risk on the basis of cohort studies.

**Methods:**

We systematically reviewed PubMed, EMBASE, Web of Science, and Chinese National Knowledge Infrastructure databases for relevant cohort studies up to May 1, 2019. Pooled hazard ratios (HRs) with 95% confidence intervals (CIs) for total and subgroup analyses were calculated with Stata 12.0 software for the assessment of the relationship between APRI and HCC risk.

**Results:**

A total of 13 studies, involving 8897 patients, were included in the meta-analysis, of which 11 explored the association between pretreatment APRI and HCC risk and four reported the relationship between posttreatment APRI and HCC risk. Pooled results showed that an elevated level of pretreatment APRI was associated with increased HCC risk (HR = 2.56, 95% CI: 1.78–3.68). When stratified by hepatitis type, high pretreatment APRI predicted HCC development in patients with chronic hepatitis B (CHB) and C (CHC) but not in alcoholic liver cirrhosis (ALC). In the subgroup analyses of study region, cut-off value, sample size, and analysis method, the relationship between high pretreatment APRI and increased HCC risk was significant. Meanwhile, patients with a high level of posttreatment APRI suffered from high HCC risk (HR = 3.69, 95% CI: 2.52–5.42). *Conclusion*: Results revealed a significant association between elevated APRI and HCC development in patients with chronic hepatitis, suggesting that APRI might serve as a valuable predictor for HCC risk in patients with chronic hepatitis.

## 1. Introduction

Hepatocellular carcinoma (HCC) is the second most common cause of cancer-related deaths worldwide [1]. Globally, approximately two billion people are infected with hepatitis B virus (HBV) and 71 million people are infected with hepatitis C virus (HCV) which are thought to be the important causes of HCC [2, 3]. The development of HCC is also associated with chronic liver disease caused by alcohol consumption and metabolic syndrome [4]. Among patients diagnosed with early stage HCC, tumor resection and liver transplantation improve prognosis with a five-year survival rate of more than 30% [5]. However, many patients with HCC are already in advanced disease during diagnosis and have poor prognosis with median survival of less than a year and a five-year survival rate of approximately 10% [6, 7]. Therefore, risk prediction and early HCC diagnosis should be given utmost attention in patients with chronic hepatitis.

Patients with advanced fibrosis and cirrhosis are at high risk of developing HCC [8, 9]. Liver biopsy has been considered as the golden standard for determining the extent of fibrosis and cirrhosis. However, liver biopsy is not widely used in clinical practice because of its invasive nature and potential complications; thus, noninvasive methods for the evaluation of liver fibrosis and cirrhosis have been developed [10]. Several serum markers, such as aspartate aminotransferase- (AST-) to-platelet ratio index (APRI), AST/alanine aminotransferase, platelets, AST, albumin, total bilirubin, and alkaline phosphatase, which are changeable depending on disease activities representing different clinical significance, have been investigated to assess liver fibrosis and cirrhosis [11, 12].

APRI showed higher accuracy than other serum markers in predicting advanced fibrosis and cirrhosis in patients with chronic hepatitis and significantly differentiated the F3 and F4 stages [13]. According to the guideline of the Asian Pacific Association for the Study of the Liver, APRI score is the most cost-effective noninvasive tool for assessing cirrhosis and active hepatitis, which may even be an alternative to liver biopsy [14]. Recently, studies have investigated whether APRI can assess the risk of HCC in patients with chronic hepatitis [15–27]. However, results have been inconsistent. Herein, to further clarify the relationship between APRI and the risk of HCC, we performed a systematic review and meta-analysis using available evidence from cohort studies.

## 2. Materials and Methods

### 2.1. Search Strategy

This meta-analysis was conducted in accordance to the Preferred Reporting Items for Systematic Reviews and Meta-Analyses. We comprehensively searched the electronic databases of PubMed, EMBASE, Web of Science, and Chinese National Knowledge Infrastructure for updates until May 1, 2019 to assess the relationship between APRI and HCC risk in patients with chronic hepatitis. Combinations of medical subject heading terms and text words ((“aspartate aminotransferase-to-platelet ratio index” or “APRI”), and (“chronic hepatitis”) and (“hepatocellular carcinoma” or “HCC”)) were used in the searches. In addition, we manually searched the records of identified articles to further identify potential studies.

### 2.2. Inclusion and Exclusion Criteria

Literature search, study selection, and validation were systematically conducted by two independent reviewers (CMZ and JYW) who were blinded to authors and publication years. The reviewers addressed disagreements by discussion, and the arbitrator (JY) adjudicated any unsolved disagreement. Studies were selected for the meta-analysis according to the following criteria: (1) cohort studies investigated the association between APRI and the risk of HCC in chronic hepatitis regardless of the testing time; (2) literature provided sufficient data for the extraction or calculation of the hazard ratio (HR); and (3) studies were written in full text. Studies were excluded according to any of the following exclusion criteria: (1) cross-sectional or case-control studies, reviews, letters, case reports, and expert opinions and (2) studies that did not provide risk estimates and 95% CIs. If multiple studies had a similar cohort of participants, only the study with the largest sample size was included for any given result [28].

### 2.3. Data Extraction and Quality Assessment

Data were independently extracted from each eligible study by two authors (CMZ and SSX), and any disagreement was resolved through consensus. The following information, if available, was recorded: first author's name, year of publication, study country, sample size, hepatitis type, therapeutic approach, follow-up period, number of HCC, and HRs with the corresponding 95% CIs. In studies where HR estimations of univariate and multivariate analyses were both provided, the results of multivariate analysis were preferentially selected. If HRs and 95% CIs were not provided directly, we attempted to estimate these points.

The Newcastle-Ottawa Scale (NOS) was applied to evaluate the quality of each included article [29]. NOS scores ranged from 0 to 9, and the study with a score of more than 6 was considered of high quality.

### 2.4. Statistical Analysis

In this meta-analysis, the relationship between APRI and HCC risk in patients with chronic hepatitis was evaluated by calculating pooled HRs and 95% CIs. An HR of >1 with 95% CIs exceeding 1 indicated an increased risk of HCC in patients with evaluated APRI. Subgroup analyses were conducted according to study region, hepatitis type, cut-off value, sample size, and analysis method.

The Chi square-based *Q* test and *I*^2^ were used in the assessment of heterogeneity between the studies. A *P* value ≤ 0.05 or *I*^2^ ≥ 50% indicated significant heterogeneity, and a random-effect model was used for the calculation of the pooled results [30]; otherwise, a fixed-effect model was selected [31]. Metaregression analysis was conducted to determine the potential source of heterogeneity. Sensitivity analysis was also conducted by omitting each study to access the stability of the results. Publication bias was estimated by visually assessing the asymmetry of the funnel plot and then quantitatively by Begg's and Egger's tests [32]. If there was significant publication bias, the trim-and-fill method was then applied to assess its influence on the dependability of the results of our meta-analysis [33]. Stata 12.0 (Stata Corporation, College Station, TX, USA) was used in the statistical analyses. All *P* values were two-tailed, and statistical significance was defined as *P* < 0.05 for all tests.

## 3. Results

### 3.1. Description of Included Studies

**Figure 1:**
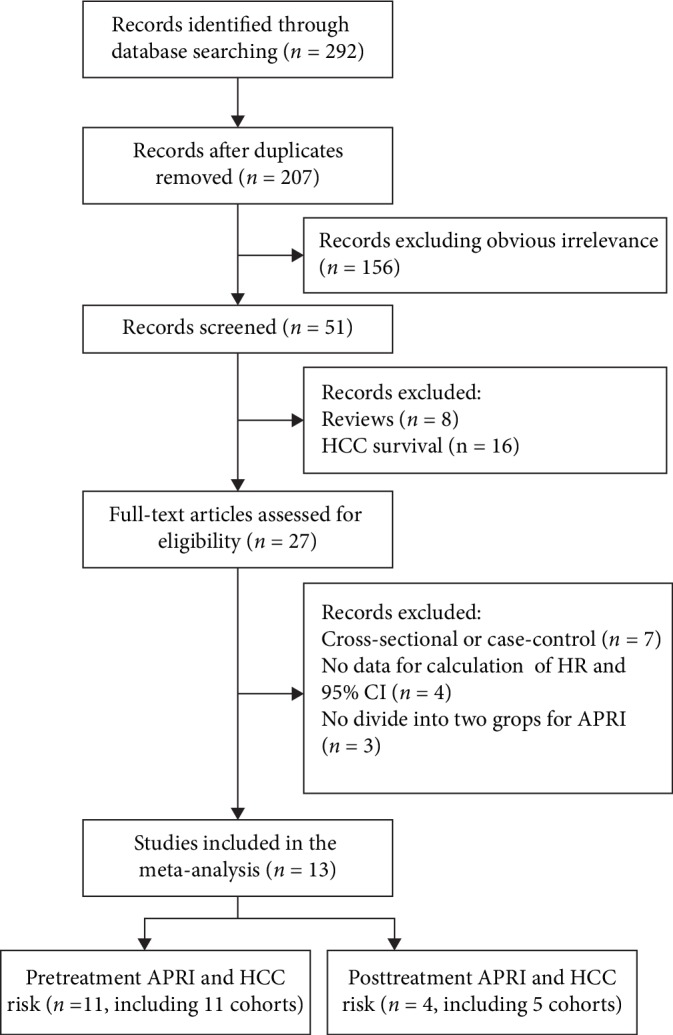
Flow diagram of the study selection process and specific reasons for exclusion in the meta-analysis.

A total of 292 records were retrieved from the databases, of which 241 papers were removed because of duplicates and apparent irrelevance. After careful review, 24 studies were further excluded, of which eight were reviews and 16 reported survival of HCC. From the full-text articles reviewed for eligibility, the following 14 papers were removed after evaluation: seven articles that were cross-sectional or case-control studies, four papers that did not provide sufficient data for HR calculation, and 3 reports that did not divide APRI into two groups. Finally, 13 studies with 16 cohorts were included [15–27], of which 11 studies with 11 cohorts reported the relationship between pretreatment APRI and HCC risk [15–25] and four papers with five cohorts reported the relationship between posttreatment APRI and HCC risk [20, 23, 26, 27]. This is because two studies reported the relationship between APRI and HCC risk before and after treatment [20, 23], and one study analyzed the association between posttreatment APRI and HCC risk in patients with chronic hepatitis C (CHC), with and without sustained virological response (SVR) separately [26]. The process of literature selection is illustrated in Figure 1.

The main characteristics of the included studies are summarized in Table 1. For pretreatment APRI, a total of 7398 patients with chronic hepatitis were included in the meta-analysis, of which 494 individuals developed HCC [15–25]. Among these 11 estimates, five were derived from patients with chronic hepatitis B (CHB) [15, 16, 19, 21, 25], five from patients with CHC [17, 20, 22–24], and only one from a patient with alcoholic liver cirrhosis (ALC) [18]. Most of the included studies were conducted in Taiwan [17, 23, 24] and Korea [15, 16, 21, 22], whereas other publications were from Japan [19], China [20], and the USA [25]. Sample sizes ranged from 34 to 1351, and the cut-off values ranged from 0.5 to 2. The HRs with 95% CIs were directly extracted from multivariate analyses in seven cohorts [15–19, 24, 25], whereas other cohorts were obtained from univariate analyses or Kaplan-Meier curves [20–23]. Meanwhile, five cohorts with 2884 patients reported the association between posttreatment APRI and HCC risk [20, 23, 26, 27]. All participants were CHC patients, and the studies were conducted in Taiwan and China.

### 3.2. Quality Assessment

The assessment of methodological quality of the included studies is shown in Table 2. On the basis of the NOS system, quality scores ranged from 6 to 8, which indicated the reliable overall quality of studies.

### 3.3. APRI and Risk of HCC

**Figure 2:**
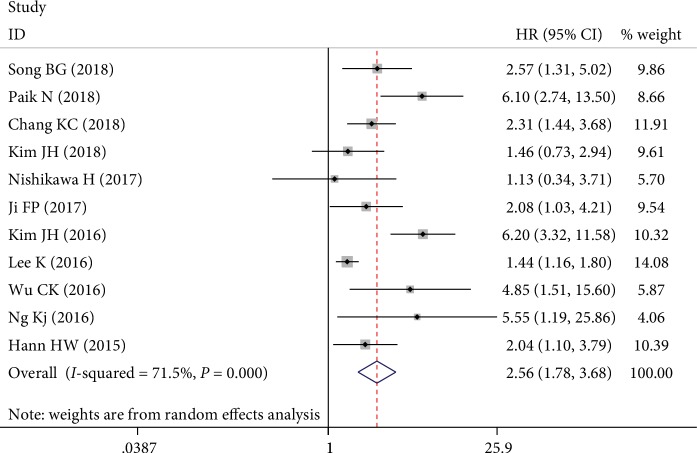
Forest plots of the overall outcomes for HCC risk of pretreatment APRI. Hazard ratios (HRs) for each trial are represented by the squares, and the horizontal lines crossing the square stand for the 95% confidence intervals (CIs). The diamonds represent the estimated pooled effect of the overall outcome for risk of HCC in patients with chronic hepatitis. All *P* values are two sided.

The main results of the analysis on the association between APRI and HCC risk are shown in Table 3. As shown in Figure 2, nine individual cohorts showed significant associations between pretreatment APRI and HCC risk, whereas the other two reported null associations. The final result showed that patients with high pretreatment APRI had increased risk of HCC with a pooled HR of 2.56 (95% CI: 1.78–3.68; *I*^2^ = 71.5%; *P*_heterogeneity_ < 0.001). When the included studies were stratified into subgroup analyses according to regions, high pretreatment APRI was associated with increased risk of HCC in Taiwan (HR = 2.71, 95% CI: 1.78–4.12, fixed effects), Korea (HR = 2.80, 95% CI: 1.44–5.46, random effects), and others (HR = 1.90, 95% CI: 1.23–2.93, fixed effects). On the basis of hepatitis type, higher APRI predicted HCC development in patients with CHB (HR = 3.16, 95% CI: 1.77–5.63, random effects) and CHC (HR = 2.17, 95% CI: 1.42–3.32, random effects) but not in patients with ALC (HR = 1.46, 95% CI: 0.73-2.94, random effects). The stratification based on the cut-off value indicated that high pretreatment APRI was still an unfavorable risk of HCC. Similarly, in the subgroup analysis of sample size, high APRI was related to increased risk of HCC not only in a large sample size (≥800) (HR = 2.79, 95% CI: 1.76–4.42, random effects) but also in a small sample size (<800) (HR = 2.37, 95% CI: 1.36–4.13, random effects). For the analysis method, the predictive role of high APRI on higher risk of HCC did not change in multivariate analysis (HR = 2.37, 95% CI: 1.82–3.09, fixed effects) and univariate analysis (HR = 2.90, 95% CI: 1.30–6.46, random effects).

**Figure 3:**
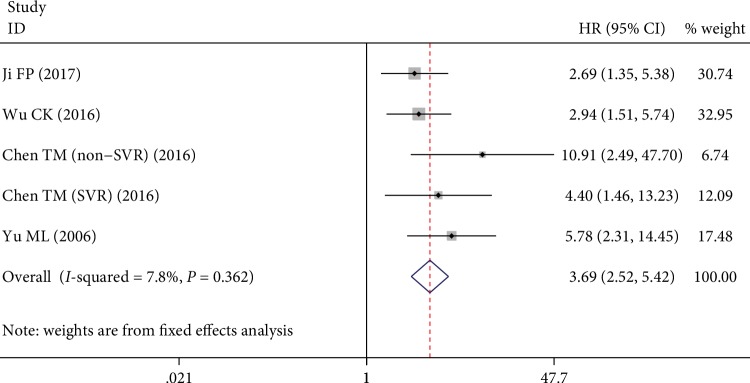
Forest plots of the overall outcomes for HCC risk of posttreatment APRI. Hazard ratios (HRs) for each trial are represented by the squares, and the horizontal lines crossing the square stand for the 95% confidence intervals (CIs). The diamonds represent the estimated pooled effect of the overall outcome for risk of HCC in patients with chronic hepatitis. All *P* values are two sided.

Additionally, four studies with five estimations evaluated the association between posttreatment APRI and risk of HCC, of which the pooled result indicated that a high level of posttreatment APRI was significantly corrected with increased risk of HCC (HR = 3.69, 95% CI 2.52–5.42, Figure 3).

### 3.4. Metaregression Analysis

Metaregression was performed to investigate the potential source of heterogeneity among studies of pretreatment APRI. Results demonstrated that study region (*P* = 0.245), hepatitis type (*P* = 0.542), cut-off value (*P* = 0.600), sample size (*P* = 0.643), and analysis method (*P* = 0.373) cannot explain the source of heterogeneity. We did not conduct metaregression analysis concerning the risk of HCC posttreatment APRI because of insufficient studies.

### 3.5. Sensitivity Analysis and Publication Bias

**Figure 4:**
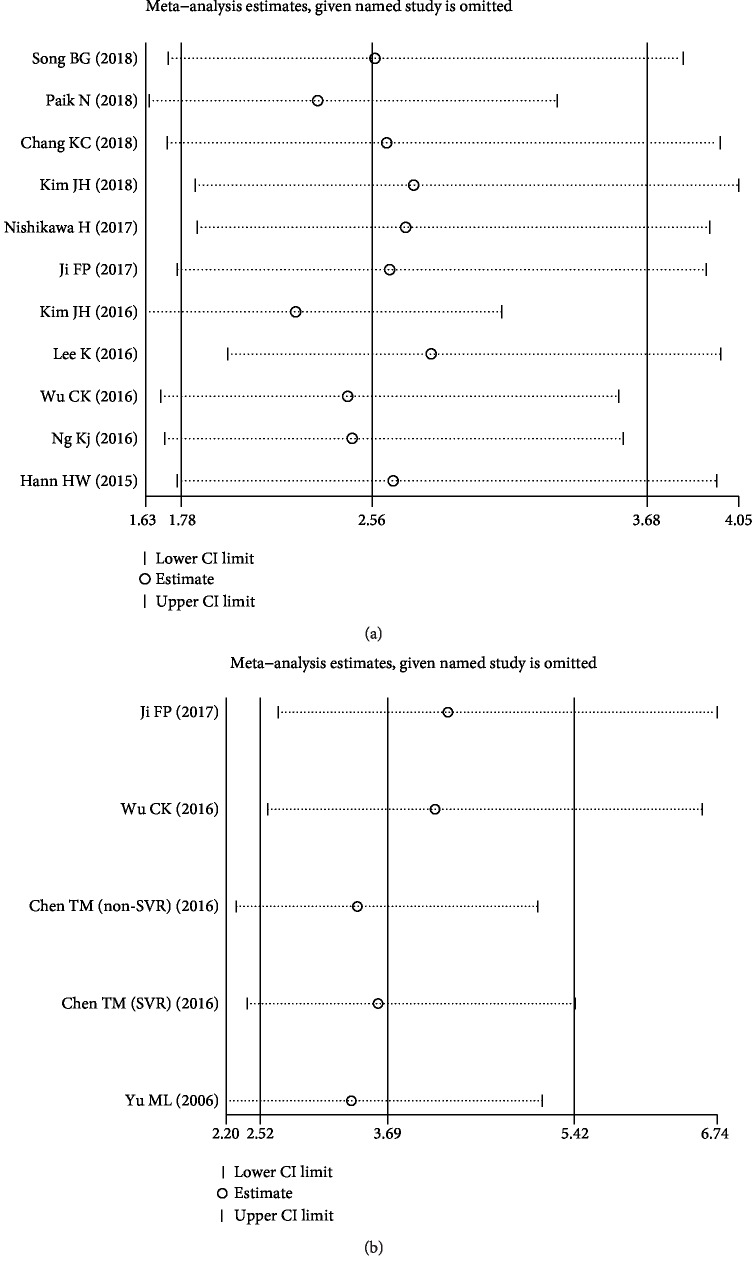
Effects of individual studies on pooled hazard ratios (HRs) for APRI and HCC risk. (a) The result of sensitivity analysis for pooled HCC risk estimation of pretreatment APRI. (b) The result of sensitivity analysis for pooled HCC risk estimation of posttreatment APRI.

Sensitivity analysis indicated that the corresponding pooled HRs for risk of HCC pretreatment (Figure 4(a)) or posttreatment (Figure 4(b)) APRI did not significantly change by ignoring any signal cohort study. Thus, results of our meta-analysis were stable.

**Figure 5:**
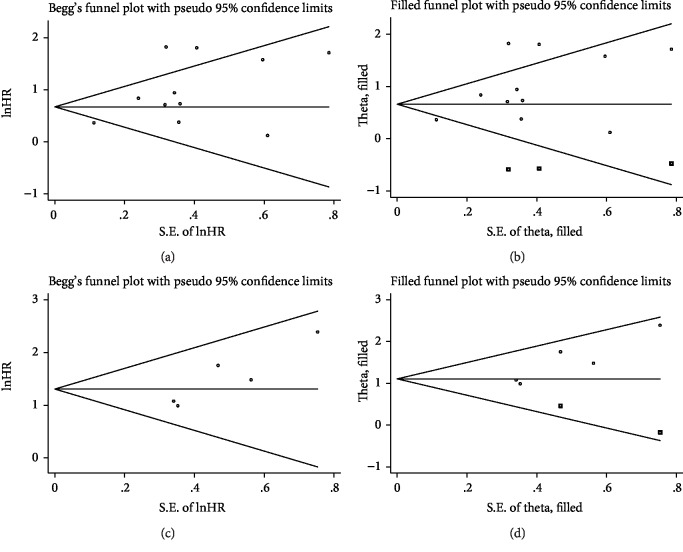
Begg's funnel plots assessing potential publication bias in studies of APRI in patients with chronic hepatitis. Each study is represented by one circle. The horizontal line represents the pooled effect estimate. (a) Funnel plot of publication bias for studies reporting risk of HCC pretreatment APRI. (b) Funnel plot adjusted with trim-and-fill methods for studies reporting risk of HCC pretreatment APRI. (c) Funnel plot of publication bias for studies reporting risk of HCC posttreatment APRI. (d) Funnel plot adjusted with trim-and-fill methods for studies reporting risk of HCC posttreatment APRI.

Although Begg's test (*P* = 0.533) did not show significant publication bias concerning pretreatment APRI, a significant publication bias was found by Egger's test (*P* = 0.039). Additionally, the funnel plot showed a certain degree of apparent asymmetry (Figure 5(a)). After adjustment using trim-and-fill analysis, three nonpublished studies were added to balance the funnel plot (Figure 5(b)). The recalculated HR and 95% CI were slightly changed but remain significant (HR = 1.94, 95% CI: 1.32–2.83), indicating the robustness of the results. As for the HCC risk of posttreatment APRI, a significant publication bias was observed by Egger's test (*P* = 0.031) but not by Begg's test (*P* = 0.221), which was also confirmed by the funnel-plot shape (Figure 5(c)). After adjusted by the trim-and-fill analysis, two more studies were needed to add into the funnel plot (Figure 5(d)) and the recalculated results did not significantly change (HR = 3.03, 95% CI: 2.14–4.27), indicating that potential publication bias had minimal impact on the overall outcome.

## 4. Discussion

As a simple and noninvasive marker, APRI consists of two routinely available biochemical and clinical parameters, aspartate aminotransferase (AST) and platelets (PLT), which can be calculated according to the following formula: (AST/the upper limit of normal value)/PLT (10^9^/L) × 100 [34]. Thus, APRI, as a composite biomarker, is less expensive and widely available [16, 35, 36] and its clinical value can be enhanced due to its stability and reliability [28]. APRI was originally proposed to predict the degree of liver fibrosis stage and liver function reserve [37, 38]. The liver fibrosis stage is one of the main factors affecting treatment decision for patients with chronic hepatitis. The main reason is that liver disease progresses more slowly in patients without fibrosis or with minimal fibrosis, and treatment does not necessarily need to be started for these patients. In contrast, patients with moderate or severe fibrosis should be treated timely because of the risk of evolution from cirrhosis to HCC and its associated complications. Liver biopsy is currently regarded as the gold standard for the extent of liver fibrosis in patients with chronic hepatitis. However, biopsy is an invasive procedure with potential complications, and sampling error can result in substantial misdiagnosis and staging inaccuracies, which lead to the development of noninvasive methods for the evaluation of liver fibrosis and cirrhosis. Borsoi Viana et al. reported that APRI has satisfactory sensitivity and specificity together with a high predictive value for the evolution of CHC [39]. Kurger et al. found that APRI was a simple bedside marker for advanced fibrosis that can avoid liver biopsy in patients with nonalcoholic steatohepatitis [40]. Therefore, APRI can be an alternative to liver biopsy to evaluate liver fibrosis status.

Liver fibrosis is closely associated with HCC, and liver cirrhosis is an important risk factor for the development of HCC [41, 42]. APRI, as a reliable index for liver fibrosis, may be a prospective predictor of HCC risk in patients with chronic hepatitis. Paik et al. reported that patients with high APRI had higher risk of developing HCC [16], whereas Kim et al. showed that there was no statistical difference in the prediction of HCC development by APRI [18]. Given the extreme inconsistency of the results, the predictive value of APRI for the occurrence of HCC in patients with chronic hepatitis is still controversial. Thus, we conducted the meta-analysis to evaluate the association between APRI and the risk of HCC in patients with chronic hepatitis.

We included 13 studies with 16 cohorts in the meta-analysis to explore the relationship between APRI and HCC risk. In the pooled results of 11 individual studies, patients with high pretreatment APRI have a high risk of HCC with a pooled HR of 2.56 (95% CI: 1.78–3.68). On the basis of subgroup analyses results, the overall outcomes did not change significantly in terms of the grouping of study region, cut-off value, sample size, and analysis method. However, when groups were based on hepatitis type, the relationship between a high level of pretreatment APRI and an increased risk of HCC remained significant in patients with CHB and CHC but not in patients with ALC. However, because of the small sample size, this result should be explained in caution. Thus, additional articles involving ALC patients are required to consolidate or overthrow the conclusion. However, chronic hepatitis had increased platelets and decreased AST after treatment and baseline APRI before treatment, which may be less precise than posttreatment APRI [23, 43]. In the present study, studies reported that dynamic APRI or posttreatment APRI helped to predict HCC development [20, 23, 26, 27]. For posttreatment APRI, the pooled HR estimate (HR = 3.69, 95% CI 2.52–5.42) based on four studies with five cohorts indicated that high posttreatment APRI corresponded to an increased risk of HCC. Therefore, APRI may serve as an independent negative biomarker for HCC events in patients with chronic hepatitis and may guide clinical strategies for enhancing the effectiveness of treatments for patients with chronic hepatitis. Patients with chronic hepatitis, who had consistently high APRI before and after treatment, had the highest incidence of HCC. Patients with reduced APRI after treatment had a decreased risk of HCC. Patients with sustained low APRI before and after treatment had the lowest risk. Clinical physicians may follow this individualized HCC surveillance strategy accordingly [23].

Although sensitivity analyses have confirmed the robustness of the pooled results, there was moderate to extreme heterogeneity between studies for HCC risk of pretreatment APRI. Thus, metaregression was performed to find the potential source of heterogeneity among studies. However, none of these factors were able to explain the heterogeneity. Given the significant publication bias among the studies concerning the risk of HCC pretreatment and posttreatment APRI (*P* < 0.05), which may have inflated overall results, trim-and-fill analyses were performed for the recalculation of the pooled results. Moreover, the adjusted HRs and 95% CIs were slightly changed but remained statistically significant, which indicated that publication bias had limited influence on the pooled findings. Therefore, the results of this meta-analysis are robust and reliable.

The mechanism of high APRI that increases the risk of HCC remains unclear. However, there are some possible explanations. First, the AST component of APRI can be elevated because of liver stress or damage caused by liver cirrhosis: clearance of AST may have been impaired, with the release of AST from injured mitochondria, which are the main factors leading to HCC [44, 45]. Meanwhile, Sanad et al. noted that the average levels of AST increased with the degree of fibrosis and further increased in cases of HCC [46]. In addition, a parallel relationship between the serum level of AST activity and the degree of liver disease in Egyptian patients with HCV infection has been reported [47, 48]. In contrast, the PLT component of APRI can be decreased because of progressive liver fibrosis and the progressive destruction of an enlarged spleen [49, 50]. Meanwhile, it has been well documented that the PLT level decreased with the progression of liver fibrosis [51]. Therefore, taken together, APRI is closely linked to the risk of HCC in patients with chronic hepatitis.

Several limitations must be taken into account. First, the current meta-analysis mainly focused on an Asian population, and the results may not be applied to other populations. Second, the cut-off values for defining high or low APRI were inconsistent, which might cause heterogeneity to some extent. Third, all the included cohort studies were retrospective, except for the study by Yu et al. [27], which was a bidirectional cohort study. Therefore, more prospective studies are required for the assessment of the relationship between APRI and HCC risk in patients with chronic hepatitis. Third, some individual HR estimations were obtained from univariate analysis and were not adjusted for potential confounders, which might have caused bias toward the overestimation of the effect size. Some other factors may affect AST and PLT, such as antiviral drugs, which should also be noted. Finally, all the included studies were limited to published studies, and unpublished reports or ongoing studies were not included in this review.

## 5. Conclusion

The results of our meta-analysis identified a significant association between high APRI and increased risk of HCC in patients with chronic hepatitis. APRI can serve as a convenient, inexpensive, simple, and reproducible biomarker for HCC risk in patients with chronic hepatitis because of its availability as a blood routine test in daily clinical practice. In the future, larger prospective studies are required to verify and strengthen our findings.

## Figures and Tables

**Table 1:** Main characteristics of the included studies.

Study (authors-year)	Region	Sample size	Hepatitis type	Follow-up period (years)	No. of HCC (%)	Cut-off value	APRI high (%)	Analysis method	HR estimation (95% CI)
Pretreatment									
Song BG (2018)	Korea	1014	CHB	Median 3.9	37 (3.6)	0.5	270 (26.6)	M	2.57 (1.31-5.02)
Paik N (2018)	Korea	1006	CHB	Median 5.1	36 (3.6)	0.5	219 (21.7)	M	6.10 (2.74-13.50)
Chang KC (2018)	Taiwan	800	CHC	Median 4.46	100 (12.5)	2.57	155 (19.4)	M	2.31 (1.44-3.68)
Kim JH (2018)	Korea	924	ALC	Median 4.83	83 (9.0)	1	120 (13.0)	M	1.46 (0.73-2.94)
Nishikawa H (2017)	Japan	338	CHB	Median 4.99	33 (9.8)	0.786	188 (55.6)	M	1.13 (0.34-3.71)
Ji FP (2017)	China	34	CHC	Median 3.45	5 (14.7)	2.5	12 (35.3)	U	2.08 (1.03-4.21)
Kim JH (2016)	Korea	542	CHB	Until Sept. 2012	68 (12.5)	0.766	214 (39.5)	U	6.20 (3.32-11.58)
Lee K (2016)	Korea	598	CHC	Median 5.1	8 (1.3)	1.0	21 (3.5)	U	1.44 (1.16-1.80)
Wu CK (2016)	Taiwan	1351	CHC	Until July 2014	49(3.6)	0.7	1000 (10.0)	U	4.85 (1.51-15.60)
Ng KJ (2016)	Taiwan	105	CHC	Mean 4.38	15 (14.3)	2	52 (49.5)	M	5.55 (1.19-25.86)
Hann HW (2015)	USA	686	CHB	Median 4.37	60 (8.7)	Median	344 (50.1)	M	2.04 (1.10-3.79)
Posttreatment									
Ji FP (2017)	China	34	CHC	Median 3.45	5 (14.7)	1.5	13 (38.2)	U	2.69 (1.35-5.38)
Wu CK (2016)	Taiwan	1351	CHC	Until July 2014	49(3.6)	0.7	135 (10.0)	M	2.94 (1.51-5.74)
Chen TM (non-SVR) (2016)	Taiwan	183	CHC	Median 3.07	14 (7.7)	1.5	45 (24.6)	M	10.91 (2.49-47.70)
Chen TM (SVR) (2016)	Taiwan	540	CHC	Median 3.45	15 (2.8)	0.5	121 (22.4)	M	4.40 (1.46-13.23)
Yu ML (2006)	Taiwan	776	CHC	Mean 4.75	41 (5.3)	0.75	176 (22.7)	M	5.78 (2.31-14.45)

CHB: chronic hepatitis B; CHC: chronic hepatitis C; ALC: alcoholic liver cirrhosis; M: multivariate; U: univariate; SVR: sustained virologic response; non-SVR: nonsustained virologic response.

**Table 2:** Methodologic quality of included studies with the NOS.

Study (authors-year)	Representativeness of the exposed cohort	Selection of the nonexposed cohort	Ascertainment of exposure	Demonstration that outcome of interest was not present at the start of the study	Comparability of cohorts on the basis of the design or analysis	Assessment of outcome	Was it followed up long enough for outcomes to occur?	Adequacy of follow-up of cohorts	Total score
Pretreatment									
Song BG (2018)	★	★	★	—	★★	★	★	★	8
Paik N (2018)	★	★	★	—	★★	★	★	★	8
Chang KC (2018)	★	★	★	—	★★	★	★	★	8
Kim JH (2018)	★	★	★	—	★	★	★	★	7
Nishikawa H (2017)	★	★	★	—	★★	★	★	★	8
Ji FP (2017)	★	★	★	—	—	★	★	★	6
Kim JH (2016)	★	★	★	—	—	★	★	★	6
Lee K (2016)	★	★	★	—	—	★	★	★	6
Wu CK (2016)	★	★	★	—	—	★	★	★	6
Ng KJ (2016)	★	★	★	—	★	★	★	★	7
Hann HW (2015)	★	★	★	—	★★	★	★	★	8
Posttreatment									
Ji FP (2017)	★	★	★	—	—	★	★	★	6
Wu CK (2016)	★	★	★	—	★★	★	★	★	8
Chen TM (non-SVR) (2016)	★	★	★	—	★★	★	★	★	8
Chen TM (SVR) (2016)	★	★	★	—	★★	★	★	★	8
Yu ML (2006)	★	★	★	—	★★	★	★	★	8

A study can be awarded a maximum of two stars for comparability of cohorts on the basis of the design or analysis. A maximum of one star can be given for other seven items.

**Table 3:** Summary of the meta-analysis results.

Categories	Trials	HR (95% CI)	*I* ^2^ (%)	*P*_*H*_	*Z*	*P*_*z*_	*P*_*m*_
Pretreatment	11 (7398)	2.56 (1.78-3.68)^*R*^	71.5	<0.001	5.06	<0.001	
Study region							0.245
Taiwan	3 (2256)	2.71 (1.78-4.12)	10.4	0.327	4.67	<0.001	
Korea	5 (4084)	2.80 (1.44-5.46)^*R*^	86.4	<0.001	3.03	0.002	
Others	3 (1058)	1.90 (1.23-2.93)	0.0	0.657	2.91	0.004	
Hepatitis type							0.542
CHB	5 (3586)	3.16 (1.77-5.63)^*R*^	67.1	0.016	3.89	<0.001	
CHC	5 (2888)	2.17 (1.42-3.32)^*R*^	57.9	0.050	3.56	<0.001	
ALC	1 (924)	1.46 (0.73-2.94)^*R*^	NA	NA	1.06	0.287	
Cut-off value							0.600
≥1.0	5 (2461)	1.62 (1.35-1.95)	36.8	0.176	5.15	<0.001	
<1.0	5 (4251)	3.79 (2.15-6.67)^*R*^	55.7	0.060	4.61	<0.001	
NR	1 (686)	2.04 (1.10-3.79)^*R*^	NA	NA	2.26	0.024	
Sample size							0.643
≥800	5 (5095)	2.79 (1.76-4.42)^*R*^	52.2	0.079	4.35	<0.001	
<800	6 (2303)	2.37 (1.36-4.13)^*R*^	77.2	0.001	3.03	0.002	
Analysis method							0.373
Multivariate	7 (4873)	2.37 (1.82-3.09)	41.2	0.116	6.36	<0.001	
Univariate	4 (2525)	2.90 (1.30-6.46)^*R*^	86.3	<0.001	2.60	0.009	
Posttreatment	5 (2884)	3.69 (2.52-5.42)	7.8	0.362	6.68	<0.001	

CHB: chronic hepatitis B; CHC: chronic hepatitis C; ALC: alcoholic liver cirrhosis; NR: none reported; *P*_*H*_: *P* value for heterogeneity based on the *Q* test; *P*_*Z*_: *P* value for statistical significance based on the *Z* test; *P*_*m*_: *P* value for statistical outcome based on multivariate metaregression analysis; ^*R*^: random-effect model.

## Data Availability

The data supporting this meta-analysis are from previously reported studies and datasets, which have been cited. The processed data are available within the article.
